# Two new *Paratropis* Simon, 1889 (Araneae, Mygalomorphae, Paratropididae) species from Ecuador

**DOI:** 10.3897/zookeys.1286.173624

**Published:** 2026-07-30

**Authors:** Joaquim Rodrigo Moraes de Castro, Miguel Medrano, Alessandro Ponce de Leão Giupponi

**Affiliations:** 1 Universidade Federal do Estado do Rio de Janeiro (UNIRIO), 22290-240, Avenida Pasteur 458, Urca, Rio de Janeiro, RJ, Brazil Laboratório de Aracnologia, Departamento de Invertebrados, Museu Nacional/Universidade Federal do Rio de Janeiro Rio de Janeiro Brazil; 2 Coleção de Artrópodes Vetores Ápteros em Saúde de Comunidades (CAVAISC), LAC-IOC-FIOCRUZ. Manguinhos, 21040-360, Rio de Janeiro, RJ, Brazil LAGE do Departamento de Ecologia, Instituto de Biociências, Universidade de São Paulo São Paulo Brazil; 3 Laboratório de Aracnologia, Departamento de Invertebrados, Museu Nacional/Universidade Federal do Rio de Janeiro (UFRJ), Rio de Janeiro, Brazil Universidade Federal do Estado do Rio de Janeiro Rio de Janeiro Brazil; 4 LAGE do Departamento de Ecologia, Instituto de Biociências, Universidade de São Paulo, São Paulo, Brazil Coleção de Artrópodes Vetores Ápteros em Saúde de Comunidades Rio de Janeiro Brazil

**Keywords:** Andean Region, bald legged spiders, cryptic, distribution, neotropics, taxonomy

## Abstract

Two new species of mygalomoph spiders of the genus *Paratropis* Simon, 1889 are described from Ecuador. With these additions, the known diversity of the genus increases from 13 to 15 species in Ecuador, and from 27 to 29 species worldwide, raising the total number of species in the Paratropididae from 47 to 49. *Paratropis barragani***sp. nov**. is the third known species endemic to the Esmeraldas province, occurring in an environment characterized by the presence of lowland humid forests in the northwest of the province. *Paratropis intag***sp. nov**. is the first species described for the Imbabura province, in a transitional region between tropical and subtropical montane forests in the Intag valley region. *Paratropis kapak* Dupérré & Tapia, 2024, *Paratropis esmeraldas* Dupérré & Tapia, 2024, *Paratropis pukallucha* Dupérré & Tapia, 2024, and *Paratropis urku* Tauber, Perafan & Peréz-Miles, 2025 are compared with the newly described species concerning ecological, geographical, and morphological characteristics between species of northern Ecuador, and a distribution map with topographic information is presented.

## Introduction

Paratropididae Simon, 1889 is a family of medium to small-sized mygalomorph spiders containing 37 known species in four genera (World Spider Catalog 2025). It is a group with poorly known habits and ecology, native to the neotropics, spanning from South America and Antilles to the south of Mexico (Valdez-Mondragón et al. 2014). They can be found under leaf litter, logs, rocks, moss, and in burrows, in the surface layers of the soil (Perafán et al. 2019), and present a scaly cuticle adapted to adhere to substrate particles (Jocqué and Dippenaar-Schoeman 2006).

Paratropidid’s phylogenetic position within the infraorder Mygalomorphae has been extensively debated and remains uncertain. Initially, the family was placed within Theraphosoidina, closer to Theraphosidae than to Barychelidae, with a division into two proposed subfamilies: Paratropidinae and Glabropelmatinae (Raven 1985). However, with the advent of molecular studies, Bond et al. (2012) proposed a new placement in which Paratropididae was more distantly related to Theraphosoidina. It is important to note, though, that this hypothesis lacks strong support, as it was based on a single representative specimen of the group (failing to capture meaningful breadth of the clade), as stated by the authors. Subsequently, a close relationship with certain clades of Nemesiidae was proposed by Garrison et al. (2016), while Wheeler et al. (2017) suggested a relationship with Ctenizidae. Yet, both studies pointed out the questionable nature of these positions due to the limited sampling in their specific analyses a concern Wheeler et al. explicitly highlighted when discussing the paraphyletic arrangement of Ctenizidae.

In Opatova et al. (2020), Paratropididae was proposed as occupying a more basal position within the Bipectina clade, not belonging to Theraphosoidina. On the other hand, in Mori and Bertani (2020) several changes were proposed to the phylogenetic organization of Mygalomorphae, including the dissolution of the subfamily Glabropelmatinae, since its only genus, *Melloina* Brignoli, 1985, was transferred to Theraphosidae by them, later transferred to Melloinidae Perafán, Montes de Oca & Pérez-Miles, 2025 (Perafán et al. 2025), and the family Paratropididae was reinstated as a sister clade to Theraphosidae and Barychelidae, in a basal position within Theraphosoidina. In both studies, Paratropididae was considered a group of uncertain phylogenetic placement, to the extent that Opatova performed two separate analyses, one including the family, and one excluding it.

Currently, the family comprises six genera and 47 described species, with nearly half of the known diversity occurring in Ecuador (47%). *Paratropis* harbors most of this diversity, with 27 described species, representing 57% of the family total. *Paratropis* has been recovered as monophyletic under several parameters and data sets of morphological data (Peñaherrera-R. et al. 2025), although the relationships between genera inside the family remain unstable. Here, we described two new species of *Paratropis* from Ecuador, increasing the diversity of the family to 29.

## Materials and methods

The specimens were stored and examined in 70% ethanol under a Leica M205 C microscope. Photos were taken with a DMC5400 camera, and measurements of body and structures were produced with the use of Leica Application Suite X (ver. 3.7.5). The map was made using the DIVA-GIS software (ver. 7.5), in conjunction with GIMP (ver. 3.0.4) image editing software.

The description format follows Perafán et al. (2019), with some modifications. All measurements presented are in millimeters (mm) unless otherwise stated. Total body length excludes chelicerae and spinnerets. Eyes were measured by their lens size. The side used for measuring the prosomal appendages, in each described specimen, was chosen based on the number of appendages present, and the number of detached appendages, due to the fragility of the material. Measurements of the non-detached legs and female palps were taken along the dorsal line of the appendages, with the use of needles to stabilize them in a straight position, and may vary from reality, due to parallax distortion. The palpal bulb measurements and illustrations were made by detaching it from the pedipalp and using an alcohol gel base in 70% ethanol to secure the structure in the desired views (dorsal, ventral, prolateral, retrolateral). The spermathecae were clarified, using lactophenol and clove oil for better observation of the structure's morphology. Spines were considered as thick, sclerotized setae, with non-translucent apex (sensu Perafán et al. 2019). The names bacilliform and spine-like are used to describe setae with a translucent rounded apex and a translucent acute apex, respectively, following the terminology used in Santos et al. (2025).

During data collection for the distribution map, it was noted that, regarding *P. kapak*, the geographic coordinates for one specimen indicated the province of Manabí, although the publication describes the known occurrence of the species as restricted to Santo Domingo. Consequently, this data point was excluded from our discussion, as it remained unclear whether the discrepancy resulted from a coordinate error or from the manner in which the locality was described in the original text.

The examined specimens are deposited in the following institutions:

**CAVAISC** Coleção de Artrópodes Vetores Ápteros de Importância em Saúde de Comunidades, Rio de Janeiro, Brazil;

**QCAZ-I** Museo de Zoología de la Pontificia Universidad Católica del Ecuador División de Invertebrados;

**MNRJ** Museu Nacional, Rio de Janeiro, Brazil.

Abbreviations of terms in text:

**ALE** anterior lateral eyes;

**AME** anterior median eyes;

**fe** femur;

**ITC** inferior tarsal claw;

**me** metatarsus;

**pa** patela;

**PLE** posterior lateral eyes;

**PLS** posterior lateral spinnerets;

**PME** posterior median eyes;

**PMS** posterior median spinnerets;

**STC** superior tarsal claw;

**ta** tarsus;

**tc** trochanter;

**ti** tibia

## Taxonomy

**Family Paratropididae Simon, 1889**

### Genus Paratropis

Taxon classificationAnimaliaAraneaeParatropididae

Simon, 1889

369A4673-25B6-5700-A95D-63FC14E9C016

#### Type species.

*Paratropis scruposa* Simon, 1889

#### Diagnosis.

Spiders from the *Paratropis* genus can be distinguished from other genera of Paratropididae by a combination of characteristics: narrow cheliceral groove, with teeth on both margins in two juxtaposed rows; the presence of a second pair of spinnerets, PMS, absent in *Anisaspis* Simon, 1892 and *Anisaspoides* F.O. Pickard-Cambridge, 1896; ITC present only on leg I, with the exceptions of *Paratropis minuscula* Almeida & de Moraes, 2022, that does not present ITC on the tarsus of leg I, *Paratropis tortue* Sherwood, Lucas & Brescovit, 2023, that presents ITC on the tarsi of legs I & II, and *Paratropis vulcanix* Santos, Gomes, Almeida, de Moraes & Bertani, 2025, that presents ITC on all tarsi; absence of abdominal pattern (except *P. rocolita* Peñaherrera-R., Sherwood, Ríos-Tamayo & Drolshagen, 2025, and *P. intag* sp. nov.) in contrast to *Alienus* Peñaherrera-R., Sherwood, Ríos-Tamayo & Drolshagen, 2025, *Stormtropis* Perafán, Galvis & Pérez-Miles, 2019, and *Inpatropis* Peñaherrera-R., Sherwood, Ríos-Tamayo & Drolshagen, 2025; males without tibial apophysis, presence of spination, and palpal bulb with a thin, very elongated and relatively straight embolus, contrary to *Stormtropis*, and differing from *Inpatropis* by the presence of eight eyes, a baso-retrolateral cymbial apophysis and, absence of an embolar keel; females differing from all other genus of the family by presenting spermathecae with multilobulated receptacles, and with or without longitudinal folds.

#### Composition.

*Paratropis amfe* Tauber, Perafán & Pérez-Miles, 2025; *Paratropis arenosa* Dupérré & Tapia, 2024; *Paratropis aurelianoi* Tauber, Perafán & Pérez-Miles, 2025; *Paratropis barragani* sp. nov.; *Paratropis calarca* Tauber, Perafán & Pérez-Miles, 2025; *Paratropis carcosita* Dupérré & Tapia, 2024; *Paratropis chami* Tauber, Perafán & Pérez-Miles, 2025; *Paratropis cryptica* Dupérré & Tapia, 2024; *Paratropis elicioi* Dupérré, 2015; *Paratropis esmeraldas* Dupérré & Tapia, 2024; *Paratropis florezi* Perafán, Galvis & Pérez-Miles; *Paratropis intag* sp. nov.; *Paratropis kapak* Dupérré & Tapia, 2024; *Paratropis lluspiosa* Tauber, Perafán & Pérez-Miles, 2025; *Paratropis macca* Tauber, Perafán & Pérez-Miles, 2025; *Paratropis nunka* Peñaherrera-R., Sherwood, Ríos-Tamayo & Drolshagen, 2025 *Paratropis otonga* Dupérré & Tapia, 2020; *Paratropis papilligera* F.O. Pickard-Cambridge, 1896; *Paratropis pristirana* Dupérré & Tapia, 2020; *Paratropis pukallucha* Dupérré & Tapia, 2024; *Paratropis rocolita* Peñaherrera-R., Sherwood, Ríos-Tamayo & Drolshagen, 2025; *Paratropis salsa* Tauber, Perafán & Pérez-Miles, 2025; *Paratropis sanguinea* Mello-Leitão, 1923, *Paratropis scruposa* Simon, 1889; *Paratropis seminermis* Caporiacco, 1955, *Paratropis tortue* Sherwood, Lucas & Brescovit, 2023; *Paratropis tuxtlensis* Valdez-Mondragón, Mendoza & Francke, 2014; *Paratropis urku* Tauber, Perafán & Pérez-Miles, 2025; *Paratropis vulcanix* Santos, Gomes, Almeida, de Moraes & Bertani, 2025.

#### Distribution.

Brazil, Colombia, Ecuador, French Guiana, Guyana, Mexico, Peru, St. Vincent, Venezuela.

### Paratropis barragani sp. nov.

Taxon classificationAnimaliaAraneaeParatropididae

6A52FAB4-4242-56CF-9B8F-CC9BCBC2D8D3

https://zoobank.org/B2BA5C55-94A8-4DCB-9C28-022418195BD4

Figs 1, 2, 5A–D, I

#### Type material.

***Holotype***. Ecuador ♂ Male; • Esmeraldas, Tundaloma ex-Lodge, near Ricaurte; 1°10'54.8"N, 78°45'06.2"W; altitude 66 m; 12–13 Aug 2019, A. Chagas Jr. & A. Giupponi leg.; (QCAZ-I 280765). ***Paratypes***. • 1 Female; same data as holotype; (QCAZ-I 280766); • 3 Females; same data as holotype; (MNRJ 16112); • 1 Male, 1 Female; same data as holotype; (CAVAISC-ARAC 028); • 1 Female, 2 Juv.; same data as holotype; (CAVAISC-ARAC 066); 1 Male, 1 Female, 1 Juv.; Esmeraldas, Tundaloma ex Lodge, near Ricaurte; 1°10'54.8"N, 78°45'06.2"W; altitude 55 m; 8–9 Feb 2014, A. Kury & A. Giupponi leg.; (CAVAISC-ARAC 104).

#### Diagnosis.

**Figure 1.**
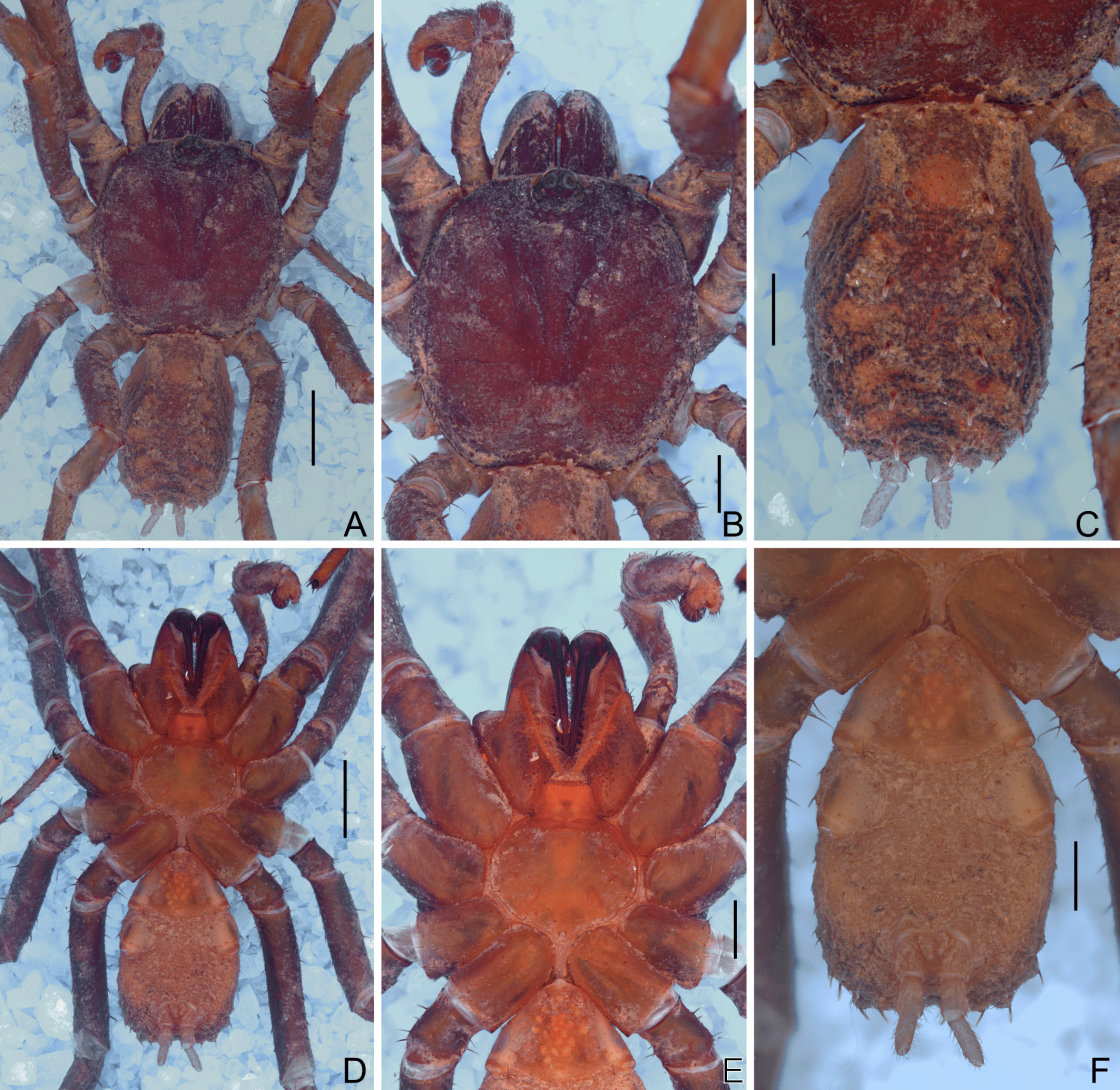
*Paratropis barragani* sp. nov. male holotype (QCAZ-I 280765). **A**. Habitus, dorsal view; **B**. Prosoma, dorsal view; **C**. Opisthosoma, dorsal view; **D**. Habitus, ventral view; **E**. Prosoma, ventral view; **F**. Opisthosoma, ventral view. Scale bars: 2 mm (**A, D**); 1 mm (**B, C, E, F**).

**Figure 2.**
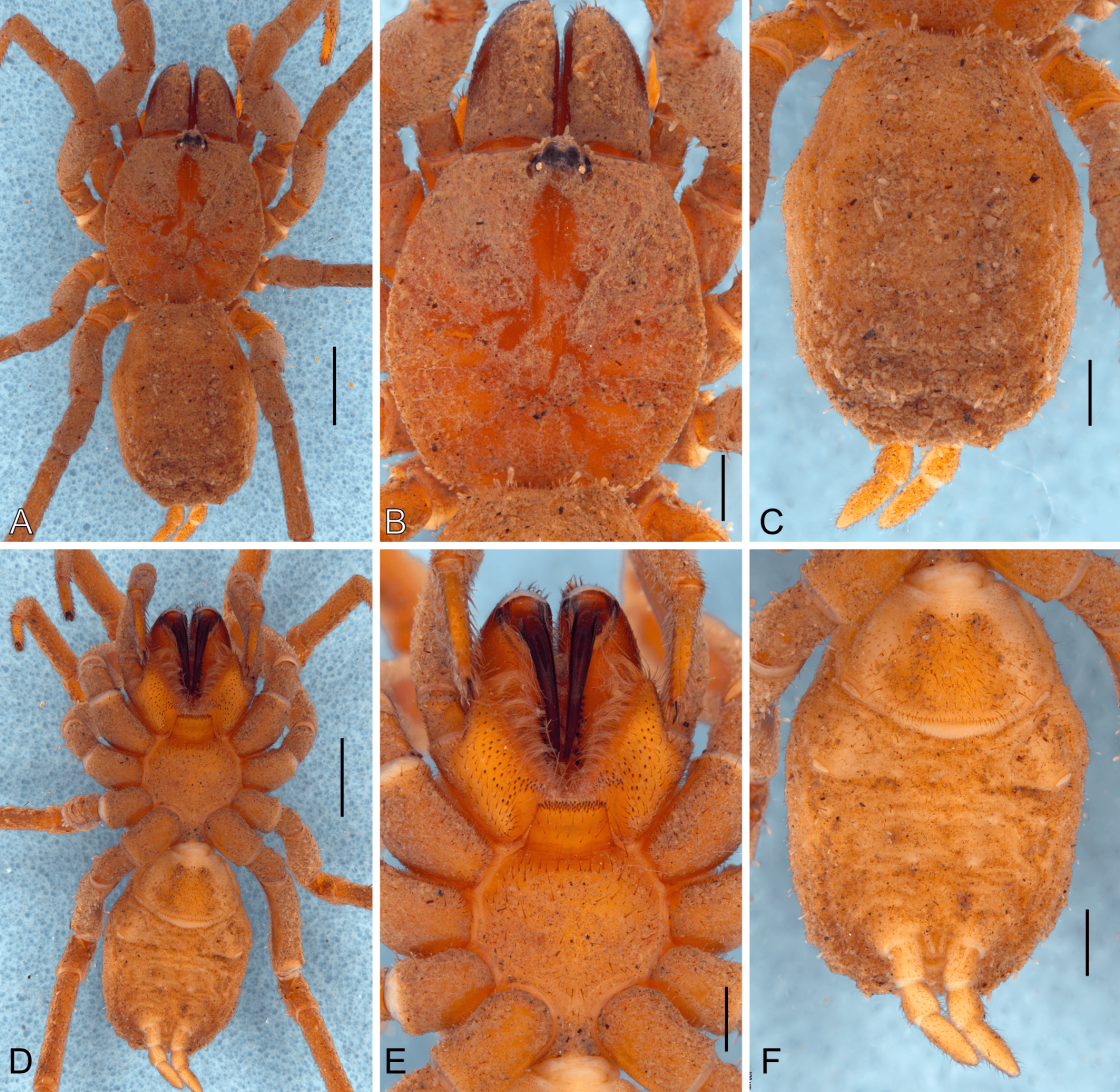
*Paratropis barragani* sp. nov. female paratype (QCAZ-I 280766). **A**. Habitus, dorsal view; **B**. Prosoma, dorsal view; **C**. Opisthosoma, dorsal view; **D**. Habitus, ventral view; **E**. Prosoma, ventral view; **F**. Opisthosoma, ventral view. Scale bars: 2 mm (**A, D**); 1 mm (**B, C, E, F**).

*Paratropis barragani* sp. nov. differs from other species of the genus by a combination of characters: opisthosoma with four dorsal rows of abdominal tubercles, each with a bacilliform macrosetae (Figs 1C, 2C); males with a straight posterior carapace margin, spine-like setae at the opisthosoma dorsal surface; and, palpal bulb with an abrupt transition to the embolus, in conjunction with a smooth curvature of the embolus tip; females with small bacilliform setae at the opisthosoma dorsal surface, spermathecae with a conical neck, narrower towards the base, and rounded head, wider than the neck, with a slight constriction. It also differs from *P. esmeraldas* because females of *P. barragani* present a setose opisthosoma; also, males of *P. esmeraldas* present a slight indent at the carapace margin, near the abdominal pedicel, which is absent in *P. barragani*.

#### Description.

Male holotype (Fig. 1). total length 10.28, carapace length 5.49, width 5.47, chelicerae length 1.65, abdomen length 4.79. Coloration (in alcohol): body with encrusted soil particles; carapace and chelicerae reddish dark brown, abdomen and legs pale brown.

***Carapace*** (Fig. 1B). Slightly setose, lateral margin with a single row of curved setae and few bacilliform setae, striae conspicuous; caput arched, shallow fovea in transversal orientation; sternum and coxae lightly bristled. Eyes and ocular tubercle: tubercle elevated, length 0.96, width 0.81, height 0.39, vaguely projected forward, with few setae. Clypeus absent. Anterior eye row slightly procurved, posterior recurved. Ocular sizes and interdistances: AME 0.32, ALE 0.26, PME 0.19, PLE 0.25, AME-AME 0.10, ALE-ALE 0.66, PME-PME 0.49, PLE-PLE 0.70. Chelicerae (Fig. 1B, E): short, with few bristles on dorsal and lateral regions, fine long bristles on ventral region. Rastellum absent. Cheliceral furrow with two continuous rows of teeth, 11/13 and 8/11 teeth on promargin and retromargin, respectively. Labium (Fig. 1E): length 0.77, width anterior edge 0.79, posterior edge 1.25, with 59 cuspules on the anterior edge. Labio-sternal groove with two lateral mounds. Maxillae longer than wide, with a prominent elongation of the anterior prolateral lobe, conical; with 63/61 cuspules, spread near the entire prolatero-ventral border, more concentrated near the proximal end. Sternum (Fig. 1E): length 2.26, width 2.74. Two pairs of oval sigilla.

***Abdomen*** (Fig. 1C, F). With four longitudinal rows of seven small tubercles, each with a bacilliform macroseta originating from its summit, from the anterior margin near the pedicel, to the posterior margin near the PLS; spine-like setae spread across the dorsum, more concentrated at the opisthosomal margin. Four book lungs apertures extrusive, oval, sclerotized. Spinnerets: length PMS 0.42, PLS 2.13, apical segment digitiform.

***Legs***. Cuticle normal. Prosomal appendices measurements provided in Table 1. Bristles, spines, bacilliform, and spine-like setae present. Trichobothria: filiform, Palp, ti 7, ta 7; leg I, ti 8, me 4, ta 7; leg II, ti 8, me 4, ta 8; leg III, ti 9, me 4, ta 7; leg IV, ti 8, me 4, ta 5. Scopula absent. Pseudoscopula weak, divided by long conical setae. Tarsal claws: ITC present on leg I; STC paired, with one medial tooth, on all legs. bacilliform setae: on all appendices, ta 0; Palp, tc 0, fe 3, pa 0, ti 0; leg I, tc 1, fe 6, pa 0, ti 0, me 0; leg II, tc 0, fe 6, pa 0, ti 2, me 0; leg III, tc 0, fe 7, pa 0, ti 0, me 0; leg IV, tc 3, fe 12, pa 3, ti 2, me 1; Spination: mainly spine-like setae on all segments. Spine: on all appendices, fe 0, pa 0; palp, ti 0, ta 0; leg I, ti 1, me 7, ta 1; leg II, ti 3, me 4, ta 1; leg III, ti 6, me 6, ta 4; leg IV, ti 4, me 4, ta 5. Leg formula: 4213.

**Table 1.** Lengths (in mm) of legs and palpal segments of male (QCAZ-I 280765) / female (QCAZ-I 280766) *Paratropis barragani* sp. nov.

	I	II	III	IV	palps
Trochanter	0.84 / 0.91	0.94 / 0.76	0.98 / 0.76	1.02 / 0.96	0.84 / 0.82
Femur	4.70 / 4.13	4.69 / 3.24	3.92 / 2.86	5.23 / 4.03	2.54 / 2.26
Patella	2.09 / 1.98	2.17 / 1.39	1.89 / 1.74	2.27 / 1.83	1.16 / 1.04
Tibia	3.52 / 3.13	3.43 / 2.30	2.70 / 2.99	4.60 / 2.83	1.78 / 1.40
Metatarsus	3.69 / 2.45	3.93 / 2.31	3.67 / 2.37	5.47 / 3.37	—
Tarsus	1.78 / 1.42	1.88 / 1.48	1.81 / 1.46	2.26 / 1.90	0.99 / 1.68
Total	16.62 / 14.02	17.04 / 11.48	14.97 / 12.18	20.85 / 14.92	7.31 / 7.20

***Palp*** (Fig. 5A–D). Cymbium with two similar lobes separated by a sclerotized groove. Palpal bulb length 0.57, pyriform, elongated. Embolus length 1.67, curving at the base, sharply on dorsal side, smoothly on ventral side, tapering towards the apex. Apex flattened, curving slightly towards the dorsum.

Female paratype (QCAZ-I 280766) (Fig. 2): total length 11.64; carapace length 5.34, width 4.76; abdomen length 6.30. Coloration (in alcohol): body with encrusted soil particles; carapace and chelicerae dark brown, legs pale brown, abdomen grayish brown.

***Carapace*** (Fig. 2B). Slightly setose, lateral margin with a line of curved setae with few disperse bacilliform setae, striae conspicuous; caput arched, shallow fovea in transversal orientation. Eyes and ocular tubercle: tubercle elevated, length 0.73, width 0.89, height 0.55, slightly projected forward, with sparse setae. Clypeus absent. Anterior eye row slightly procurved, posterior recurved. Ocular sizes and interdistances: AME 0.27, ALE 0.23, PME 0.19, PLE 0.22, AME-AME 0.14, ALE-ALE 0.65, PME-PME 0.46, PLE-PLE 0.67. Chelicerae (Fig. 2B, E): length 1.60, short bristles on dorsal area closer to the prolateral margin, few sparse fine bristles on ventral area; basal segment with bacilliform setae across its length. Rastellum absent. Cheliceral furrow with two rows of well-developed teeth, 11/12 and 9/12, on promargin and retromargin, respectively. Labium (Fig. 2E): length 0.88, width anterior edge 1.00, posterior edge 1.28, with 58 cuspules on the anterior edge. Labio-sternal groove with two lateral mounds. Maxillae longer than wide, with a prominent elongation of the anterior prolateral lobe, conical; with 51/51 cuspules, spread near the entire prolatero-ventral border, more concentrated on the proximal end. Sternum (Fig. 2E): length 2.73, width 2.01. Two pairs of oval sigilla.

***Abdomen*** (Fig. 2E, F). With four longitudinal rows of seven small tubercles, each with a bacilliform macroseta originating from its summit, from the anterior margin near the pedicel, to the posterior margin near the PLS; small bacilliform setae spread across the dorsum, more concentrated at the opisthosomal margin. Book lungs apertures extrusive, oval, sclerotized. Spinnerets: length PMS 0.46, PLS 1.76, apical segment digitiform.

***Legs***. Cuticle normal. Prosomal appendices measurements provided in Table 1. Bristles, spines, bacilliform, and spine-like setae present. Trichobothria: filiform, Palp, ti 7, ta 8; leg I, ti 8, me 4, ta 9; leg II, ti 7, me 4, ta 8; leg III, ti 7, me 4, ta 8; leg IV, ti 6, me 4, ta 8. Scopula absent. Pseudoscopula weak, divided by long conical setae. Tarsal claws: ITC present on leg I; STC paired, with one medial tooth, on all legs. Bacilliform setae: on all appendices, me 0, ta 0; Palp, tc 3, fe 5, pa 1, ti 4; leg I, tc 6, fe 13, pa 2, ti 4; leg II, tc 4, fe 14, pa 2, ti 4; leg III, tc 3, fe 10, pa 2, ti 3; leg IV, tc 6, fe 12, pa 3, ti 7; Spination: principally spine-like setae on all segments. Spines: on all appendices, tc 0, fe 0, pa 0; palp, ta 2; leg I, ti 0, me 24, ta 14; leg II, ti 4, me 7, ta 0; leg III, ti 4, me 6, ta 2; leg IV, ti 3, me 4, ta 2. Leg formula: 4132.

***Spermathecae*** (Fig. 5I). Two receptacles, neck longer than wide; neck length 0.23, width base 0.11, apex 0.14, head length 0.22, width 0.23; neck conical, wider apically; head rounded, slightly constricted.

#### Distribution.

Only known from the type locality in Ecuador, at the Ecuadorian coastal range ecosystem in the biogeographical province of the Northern Andes, in the Esmeraldas province, near Ricaurte, at 66 m altitude.

#### Etymology.

The specific epithet barragani (genitive) honors Álvaro Barragán (PUCE, Quito) for his invaluable support during field expeditions throughout Ecuador.

### Paratropis intag sp. nov.

Taxon classificationAnimaliaAraneaeParatropididae

20B3BB6A-9476-509A-9FD3-98B49C539C96

https://zoobank.org/DCCD7964-2262-4FCB-9DED-A60DB895FFA1

Figs 3, 4, 5E–H, J

#### Type material.

***Holotype*.** Ecuador ♂ 1 Male; • Imbabura, Apuela, San Antonio de Pucara, Intag Colibri lodge nature trails; 0°21'46.8"N, 78°29'09.1"W; altitude 1880 m; 2–3 Dec 2021; A. Giupponi, A. Granado, A. Kury & M. Medrano leg.; (QCAZ-I 281155). ***Paratypes***. Ecuador ♀ 1 Female; • Imbabura, Apuela, San Antonio de Pucara, Intag Colibri lodge nature trails; 0°21'46.8"N, 78°29'09.1"W; altitude 1840 m; 15–16 Jul 2019; A. Giupponi, & A. Chagas Jr.; (QCAZ-I 282400). ♂ 1 Female; • Imbabura, Apuela, San Antonio de Pucara, near Intag Colibri lodge; 0°21'54.1"N, 78°28'54.8"W; altitude 1840 m; 15–16 Jul 2019; A. Chagas jr. & A. Giupponi leg.; (MNRJ 16113); ♂ 1 Male, 1 Female, 1 Juv.; • Imbabura, Apuela, San Antonio de Pucara, Intag Colibri lodge nature trails; 0°21'46.8"N, 78°29'09.1"W; altitude 1880 m; 2–3 Dec 2021; A. Giupponi, A. Granado, A. Kury & M. Medrano leg.; (CAVAISC-ARAC 102). 1 Female; • Imbabura, Apuela, San Antonio de Pucara, near Intag Colibri lodge; 0°21'54.1"N, 78°28'54.8"W; altitude 1840 m; 15–16 Jul 2019; A. Chagas jr. & A. Giupponi leg.; (CAVAISC -ARAC 103).

#### Diagnosis.

*Paratropis intag* sp. nov. can be differentiated by presenting abdominal patterning, similar to *P. rocolita* Peñaherrera-R., Sherwood, Ríos-Tamayo & Drolshagen, 2025, in conjunction with sex-specific characteristics. Males of the species differ from others by the absence of spines on all prosomal appendages; palpal bulb thinner than that of *P. urku*Tauber et al. 2025; embolus with a median curvature towards the retrolateral side, and a distal curvature towards the dorsal side; absence of subapical triangular tooth on the embolus. Females of the species differ from others by the absence of spines on all tarsi, except that of leg I; presence of bacilliform macrosetae on the dorsal portion of all prosomal appendages, from femur to tibia; spermathecae with a long, sinuous, conical neck, wider towards the base, and a multilobed head with a small constriction and condensed vesicles, differing from *P. urku*, where the neck is short and straight from the base to its apex, and the head presents smaller sized lobes, and from *P. rocolita*, where the neck is shorter and less sinuous, while the head presents lobes in a different configuration and shape.

#### Description.

**Figure 3.**
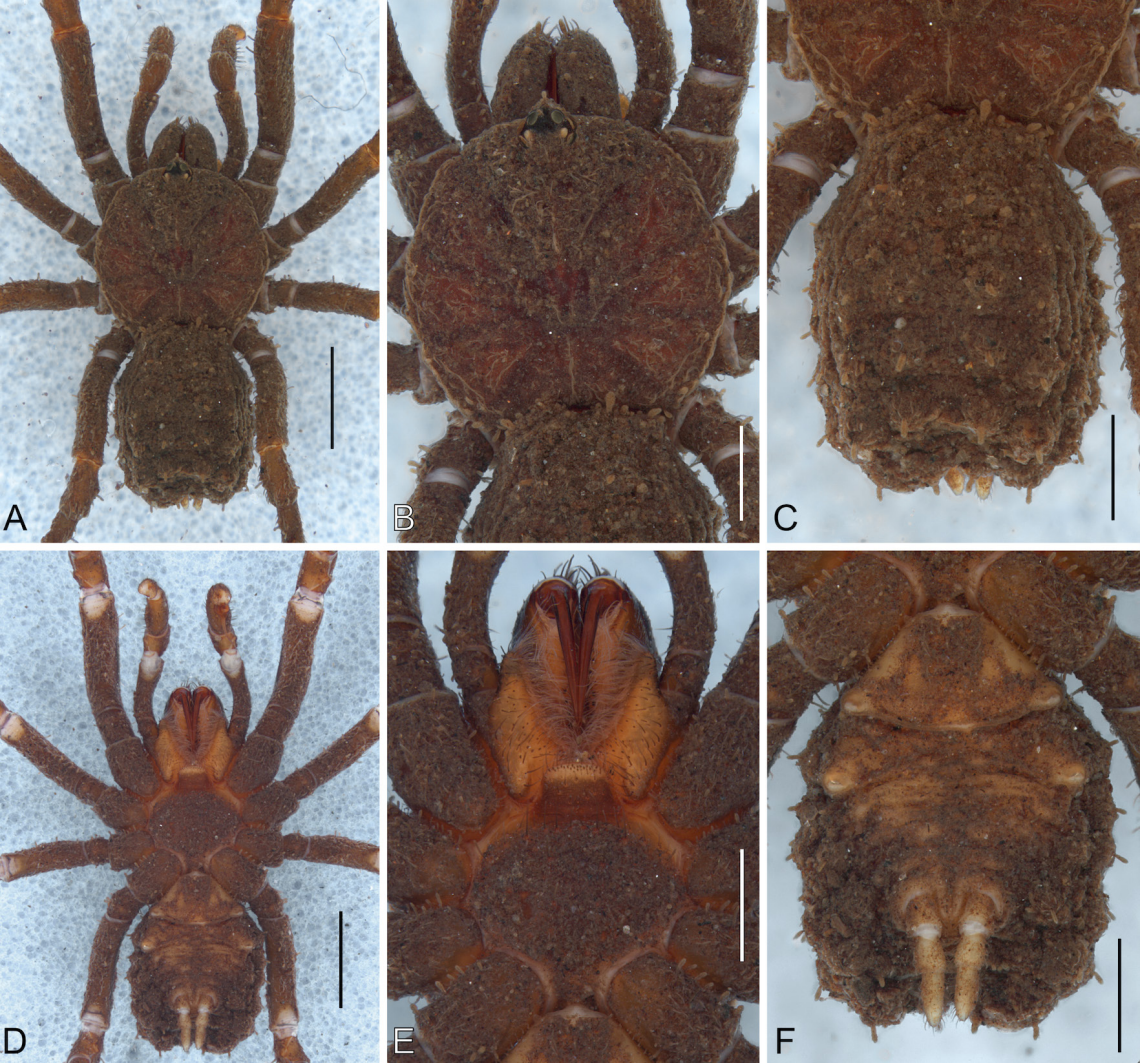
*Paratropis intag* sp. nov. male holotype (QCAZ-I 281155). **A**. Habitus, dorsal view; **B**. Prosoma, dorsal view; **C**. Opisthosoma, dorsal view; **D**. Habitus, ventral view; **E**. Prosoma, ventral view; **F**. Opisthosoma, ventral view. Scale bars: 2 mm (**A, D**); 1 mm (**B, C, E, F**).

Male holotype (QCAZ-I 281155 (Fig. 3). total length 6.51; carapace length 3.11, width 3.46; abdomen length 3.4. Coloration (in alcohol): body with encrusted soil particles; carapace and chelicerae dark brown, legs pale brown, abdomen grayish brown.

***Carapace*** (Fig. 3B). Slightly setose, lateral margin with a line of curved setae with few disperse bacilliform setae, striae conspicuous; caput arched, shallow fovea in transversal orientation. Eyes and ocular tubercle: tubercle elevated, length 0.57, width 0.59, height 0.40, slightly projected forward, with sparse setae. Clypeus absent. Anterior eye row slightly procurved, posterior recurved. Ocular sizes and interdistances: AME 0.13, ALE 0.12, PME 0.10, PLE 0.12, AME-AME 0.14, ALE-ALE 0.43, PME-PME 0.27, PLE-PLE 0.40. Chelicerae (Fig. 3B, E): length 0.91, short bristles on dorsal area closer to the prolateral margin, few sparse fine bristles on ventral area; basal segment with bacilliform setae across its length. Rastellum formed by few spine-like bristles at the distal edge of the paturon, near the base of the fangs. Cheliceral furrow with two rows of well-developed teeth, 10/9 and 4/4, on promargin and retromargin, respectively. Labium (Fig. 3E): length 0.45, width anterior edge 0.48, posterior edge 0.73, with 23 cuspules on the anterior edge. Labio-sternal groove with two lateral mounds. Maxillae longer than wide, with a prominent elongation of the anterior prolateral lobe, conical; with 25/25 cuspules, spread near the entire prolatero-ventral border, more concentrated on the proximal end. Sternum (Fig. 3E): length 1.4, width 1.94. Two pairs of oval sigilla.

***Abdomen*** (Fig. 3C, F). With six longitudinal rows of seven small tubercles, each with a bacilliform macroseta originating from its summit, from the anterior margin near the pedicel, to the posterior margin near the PLS; small bacilliform setae spread across the dorsum, more concentrated at the opisthosomal margin. Book lungs apertures extrusive, oval, sclerotized. Spinnerets: length PMS 0.24, PLS 1.15, apical segment digitiform.

***Legs***. Cuticle normal. Prosomal appendices measurements provided in Table 2. Bristles, bacilliform and spine-like setae present, spines absent. Trichobothria: filiform, Palp, ti 2, ta 2; leg I, ti 4, me 2, ta 3; leg II, ti 4, me 2, ta 3; leg III, ti 4, me 2, ta 3; leg IV, ti 3, me 2, ta 3. Scopula absent. Pseudoscopula weak, divided by long conical setae. Tarsal claws: ITC present on leg I; STC paired, with one medial tooth, on all legs. Bacilliform setae: on all appendices, ta 0; Palp, tc 6, fe 8, pa 2, ti 0; leg I, tc 5, fe 10, pa 6, ti 0, me 0; leg II, tc 5, fe 10, pa 4, ti 4, me 2; leg III, tc 3, fe 8, pa 3, ti 4, me 2; leg IV, tc 4, fe 9, pa 2, ti 4, me 1. Leg formula: 1423.

**Table 2.** Lengths (in mm) of legs and palpal segments of male (QCAZ-I 281155) / female (QCAZ-I 282400) of *Paratropis intag* sp. nov.

	**I**	**II**	**III**	**IV**	**Palp**
Trochanter	0.64/0.73	0.60/0.49	0.48/0.62	0.63/0.59	0.40/0.44
Femur	2.80/2.50	2.29/2.08	1.91/1.74	2.88/2.45	1.46/1.27
Patella	1.40/1.37	1.13/1.14	0.96/0.86	0.92/1.29	0.69/0.95
Tibia	2.20/2.03	1.46/1.40	1.19/1.16	2.04/1.78	0.99/0.83
Metatarsus	1.89/1.39	1.64/1.14	1.46/1.00	2.14/1.97	—
Tarsus	1.13/0.95	1.04/0.96	0.99/1.00	1.26/1.13	0.39/1.09
Total	10.06/8.97	8.16/7.21	6.99/6.38	9.87/9.21	3.93/4.58

***Palp*** (Fig. 5D, E). Cymbium with two similar lobes separated by a sclerotized groove. Palpal bulb length 0.29, pyriform, elongated. Embolus length 0.81, curving smoothly, towards the retrolateral side near the middle. Apex flattened, tapering towards the apex.

**Figure 4.**
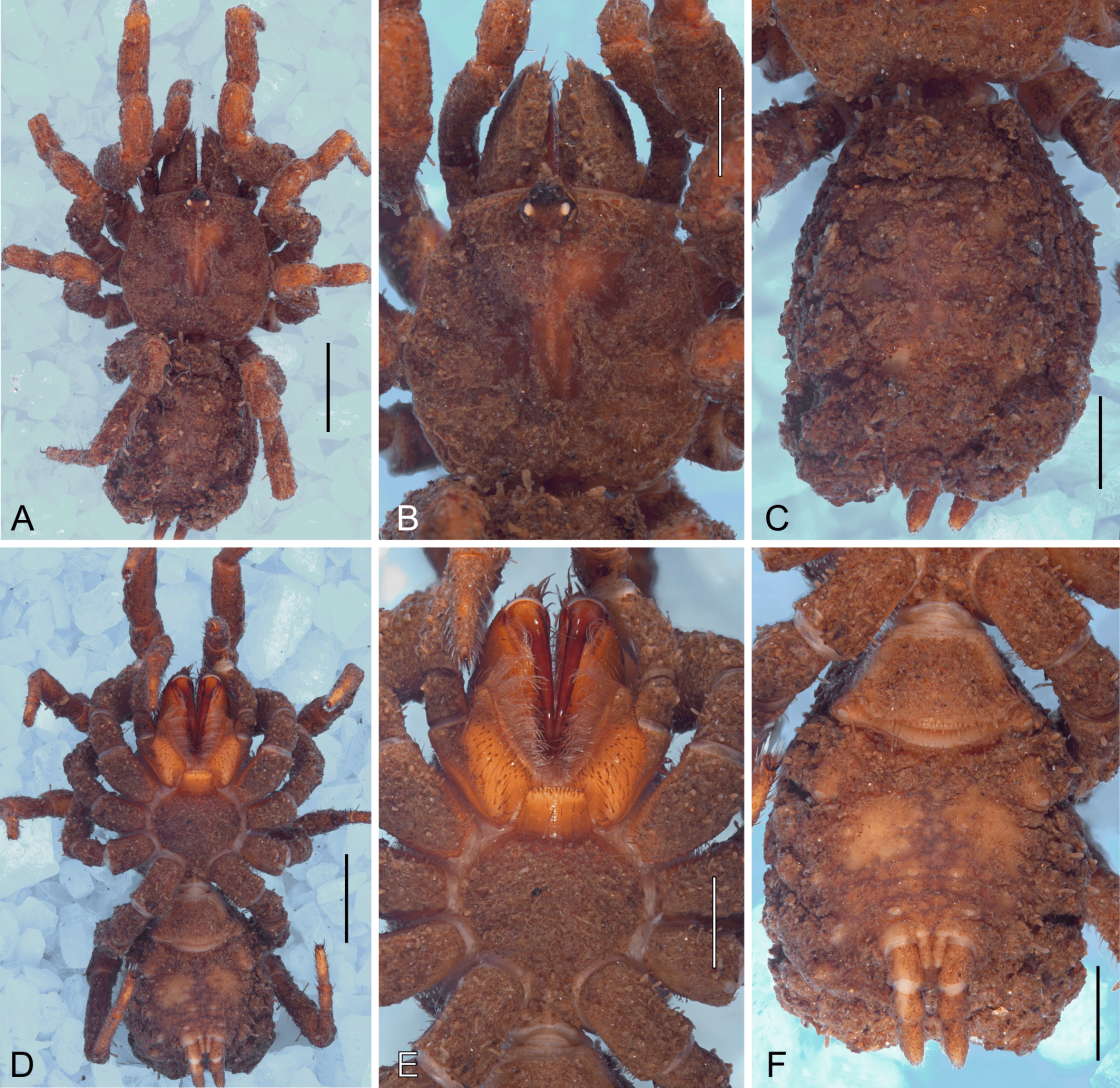
*Paratropis intag* sp. nov. female paratype (QCAZ-I 282400). **A**. Habitus, dorsal view; **B**. Prosoma, dorsal view; **C**. Opisthosoma, dorsal view; **D**. Habitus, ventral view; **E**. Prosoma, ventral view; **F**. Opisthosoma, ventral view. Scale bars: 2 mm (**A, D**); 1 mm (**B, C, E, F**).

Female paratype (Fig. 4) total length 7.75; carapace length 3.27, width 3.43; abdomen length 4.48. Coloration (in alcohol): body with encrusted soil particles; carapace, chelicerae, and legs dark brown, abdomen grayish brown.

**Figure 5.**
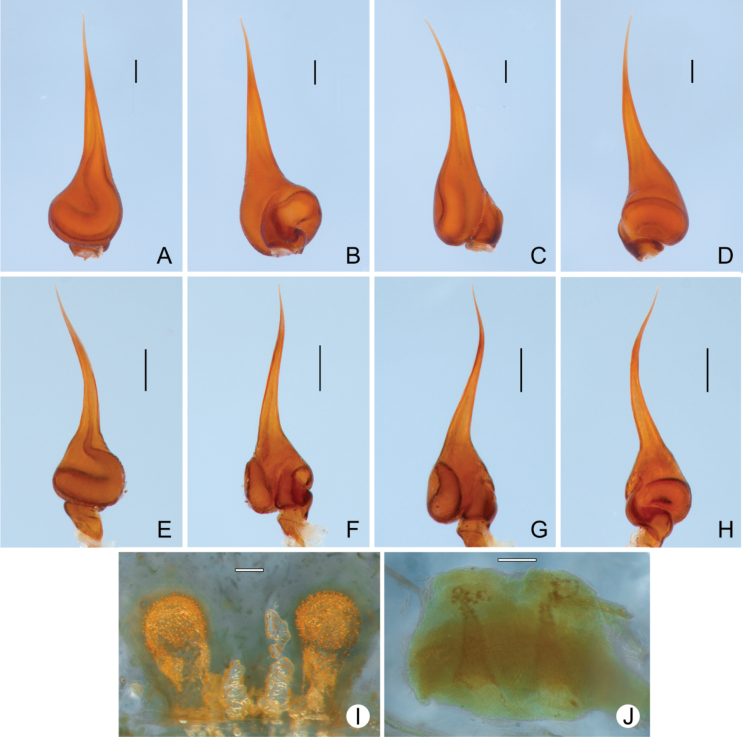
Genitalia of *Paratropis barragani* sp. nov. (**A–D, I**) and *Paratropis intag* sp. nov. (**E–H, J**). **A**. Right bulb *P. barragani* sp. nov. (QCAZ-I 280765), dorsal view; **B**. Same, ventral view; **C**. Same, prolateral view; **D**. Same, retrolateral view; **E**. Right bulb *P. intag* sp. nov. (QCAZ-I 281155) dorsal view; **F**. Same, ventral view; **G**. Same, prolateral view; **H**. Same, retrolateral view; **I**. Spermathecae of *P. barragani* sp. nov. (QCAZ-I 280766); **H**. Spermathecae of *P. intag* sp. nov. (QCAZ-I 282400). Scale bars: 200 µm (**A–H**), 100 µm (**I–J**).

***Carapace*** (Fig. 4B). Slightly setose, lateral margin with a line of curved setae, striae conspicuous; caput arched, shallow fovea in transversal orientation. Eyes and ocular tubercle: tubercle elevated, length 0.75, width 0.69, height 0.32, slightly projected forward, with sparse setae. Clypeus absent. Anterior eye row procurved, posterior recurved. Ocular sizes and interdistances: AME 0.13, ALE 0.17, PME 0.14, PLE 0.17, AME-AME 0.15, ALE-ALE 0.44, PME-PME 0.29, PLE-PLE 0.47. Chelicerae (Fig. 4B, E): length 2.02, one row of bristles and bacilliform setae near the dorsal prolateral margin, few sparse bristles on ventral area. Rastellum formed by a few long spine-like bristles at the distal edge of the paturon, near the base of the fangs. Cheliceral furrow with two rows of well-developed teeth, 9/11 and 5/5, on promargin and retromargin, respectively. Labium (Fig. 4E): length 0.55, width anterior edge 0.65, posterior edge 0.88, with 24 cuspules on the anterior edge. Labio-sternal groove with two lateral mounds. Maxillae longer than wide, with a prominent elongation of the anterior prolateral lobe, conical; with 26/27 cuspules, spread near the entire prolatero-ventral border, more concentrated on the proximal end. Sternum (Fig. 4E): length 1.53, width 2.01. Two pairs of oval sigilla.

***Abdomen*** (Fig. 4C, F). With six longitudinal rows of seven bacilliform macrosetae, from the anterior margin near the pedicel, to the posterior margin near the PLS; bacilliform setae spread across the dorsum, more concentrated at the opisthosomal margin. Book lungs apertures extrusive, oval, sclerotized. Spinnerets: length PMS 0.35, PLS 1.27, apical segment digitiform.

***Legs***. Cuticle normal. Prosomal appendices measurements provided in Table 2. Bristles and long bacilliform setae present. Trichobothria: filiform, Palp, ti 2, ta 2; leg I, ti 5, me 2, ta 4; leg II, ti 4, me 2, ta 3; leg III, ti 4, me 2, ta 3; leg IV, ti 3, me 2, ta 3. Scopula absent. Pseudoscopula weak, divided by long conical setae. Tarsal claws: ITC present on leg I; STC paired, with one medial tooth on all legs. Bacilliform setae: palp, tc 4, fe 8, pa 3, ti 3, ta 0; leg I, tc 7, fe 10, pa 1, ti 4, me 2, ta 4; leg II, tc 4, fe 8, pa 4, ti 6, me 5, ta 0; leg III, tc 5, fe 6, pa 2, ti 7, me 4, ta 0; leg IV, tc 6, fe 9, pa 2, ti 5, me 0, ta 0; Spination: few spine-like setae on most segments. Spines: on all appendices, tc 0, fe 0, pa 0; palp, ti 0, ta 0; leg I, ti 2, me 16, ta 10; leg II, ti 2, me 5, ta 0; leg III, ti 3, me 6, ta 0; leg IV, ti 3, me 6, ta 0. Leg formula: 4123.

***Spermathecae*** (Fig. 5J). Two receptacles, neck longer than wide; neck length 0.19, width base 0.11, apex 0.03, head length 0.07, width 0.09; neck conical, wider at the base, curved near the apex; head heavily constricted, multilobed, with vesicles present across its surface.

#### Distribution.

Only known from the type locality in Ecuador, at the Northern Andean Montane Evergreen Shrubland in the biogeographical province of the Northern Andes in the Imbabura province, near the city of San Antonio de Pucará, approximately 1800 m of elevation.

#### Etymology.

The specific epithet is a noun in apposition referring to the Intag valley region, from which the species was first collected.

## Discussion

Ecuador exhibits high rates of endemism and speciation, likely as a consequence of habitat diversification caused by the presence of the Andean Mountain Range (Luebert, Weigend 2014). This mountain system induces radical changes beyond elevation (maximum of 6,310 m) in temperature and humidity, and traverses Ecuador from north to south. Dupérré and Tapia (2025) discussed how this phenomenon is evident across various groups of Mygalomorphae. Thus, the distribution of Paratropididae species shows a pronounced degree of endemism, with localities separated by only a few kilometers hosting distinct species.

Although Esmeraldas houses several known Paratripididae, this region is far from homogeneous, the same applies for the biodiversity in each biogeographic province present there. In the northwest, within the Chocó-Darién biogeographic province and its lowland humid forests (0–66 m), *P. barragani* sp. nov. has been recorded. On the southwestern coast (sea level), vegetation formations are less humid and less dense, forming a transitional zone toward the drier physiognomies typical of the equatorial Pacific, in the Western Ecuador province (Morrone 2014), where *P. esmeraldas* occurs. In the south-central part, a plain influenced by Andean forest formations (100–500 m) and characterized by a more humid climate than the southern coast supports the presence of *P. pukallucha*. And at elevations of 500–800 m in the province of Santo Domingo, at the Cauca biogeographic province (Morrone 2014), *P. kapak* occurs in dense humid forest formations and a milder climate. It is relevant to consider that, although Santo Domingo province has some species recorded (such as *P. kapak*, *P. pulkallucha*, and *P. llustiosa*), they have not yet been documented co-occurring at the same specific locality. Given the substantial increase in the number of *Paratropis* species described from Ecuador over the last decade (Perafán et al. 2019; Dupérré, Tapia 2024: Peñaherrera-R et al. 2025) together with the group's high degree of endemism (Dupérré and Tapia 2024; Tauber et al. 2025), further sampling is expected to reveal additional species and better understanding of their overall distribution; however, for now, it is difficult to assume sympatry.

Two species of *Paratropis* stricto sensu from the Pichincha province were considered for the comparison with *P. intag* sp. nov., *P. urku* and *P. rocolita*. *Paratropis pasochoa* was not considered as it presents a drastically different spermathecal morphology, having a globular, uni-lobular head, which differs from the latest diagnostic definition of the genus, proposed in Peñaherrera-R et al. (2025). At approximately 1,800 m, on the western slopes of the Andes in the province of Imbabura, a transitional zone between tropical and subtropical montane forests harbors *P. intag* sp. nov.; while *P. urku* and *P. rocolita* occur at higher elevations in Pichincha (Cauca biogeographic province), at elevations of 3,600 m and 2,200 m, respectively. It is important to note that the wide elevation range of occurrence of paratropidids may indicate either physiological plasticity or reflect their cryptic habits and dependence on specific microhabitats occurring across the altitudinal gradient.

**Figure 6.**
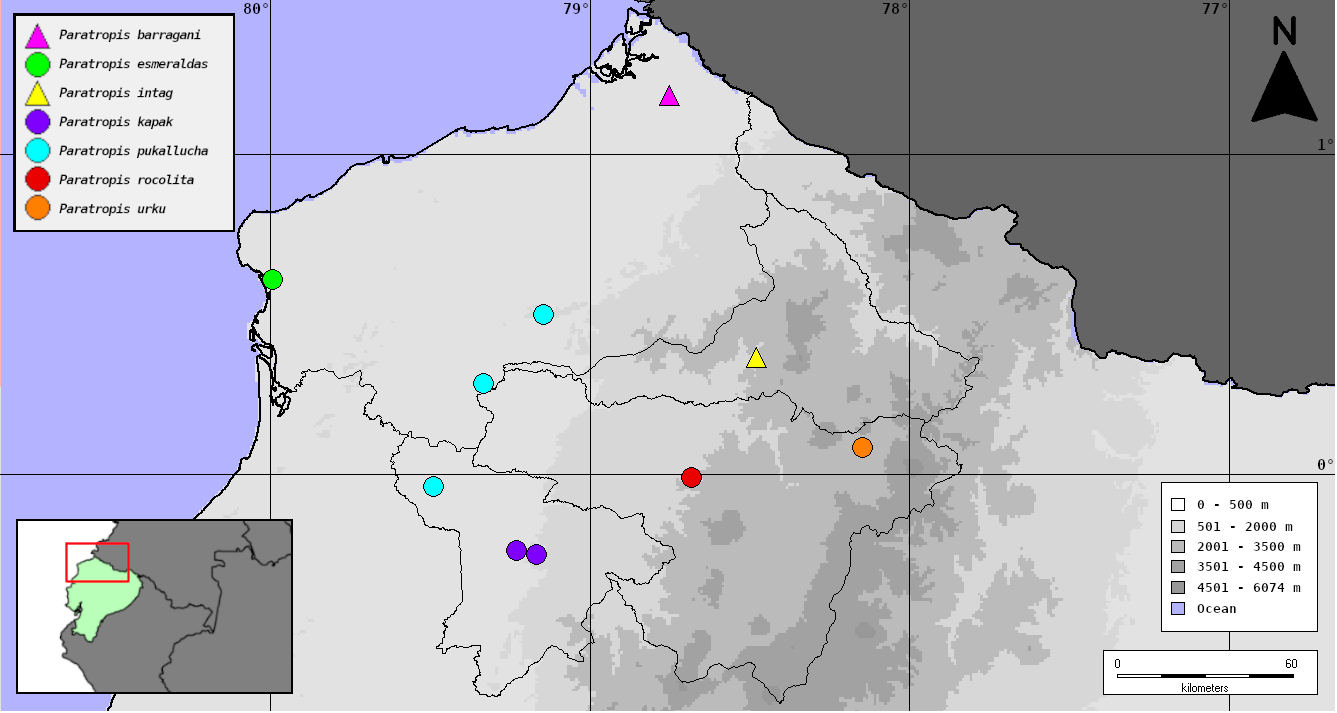
Distribution map of discussed *Paratropis* species. Scale bar: 60 km.

Despite their geographic proximity (Fig. 6), *P. barragani* sp. nov. differs from *P. esmeraldas* primarily in that females possess abdominal tubercles and setae (Fig. 2C), which are absent in *P. esmeraldas*. Additionally, the male of *P. barragani* exhibits a straight posterior margin of the carapace (Fig. 1B), whereas the male of *P. esmeraldas* displays an indentation near the abdominal pedicel. In comparison with other endemic species of the province, *P. pukallucha* and *P. kapak*, *P. barragani* sp. nov. can be distinguished by the morphology of its genitalia. The male palp, in retrolateral view (Fig. 5D), presents a more abrupt transition between bulb and embolus, along with a more curvilinear embolus (Fig. 5A–D). The female spermatheca differs primarily in the relative width between its neck and head, with the head being significantly wider than the neck (Fig. 5I). These morphological distinctions reinforce the status of *P. barragani* sp. nov. as a distinct species and corroborate the ecological differentiation among the physiognomies the species inhabits. The abovementioned species does not fully fit the diagnosis of *Paratropis* proposed by Peñaherrera-R et al. (2025), who mention the forthcoming description of a new genus of Paratropididae that could include these species. *Paratropis barragani* seems to be related to them and as it does not match the diagnosis of any currently described genera, we provisionally assign *P. barragani* to *Paratropis* until further evidence supporting the recognition of the new genus becomes available.

*Paratropis intag* sp. nov. represents the first record of the genus in the province of Imbabura, and is distinguishable by the macrosetae pattern presented on its abdomen (Figs 3C, 4C) and legs, from the femur to tibia segments of all prosomal appendages, in conjunction with sex-specific morphological characteristics. The female of the species possesses spines on the tibia and metatarsus of all legs, and on all tarsus except that of leg I, a feature shared with *P. urku*, while differentiated by the spermathecal neck size and shape, where *P. intag* presents a long neck with a curvature near its head (Fig. 5J), while *P. urku* displays a short straight neck. As for the male, it can be differentiated, especially from *P. urku* morphologically, by presenting a palpal bulb with a thinner profile (Fig. 5E–H) than that of *P. urku* and an absence of spines on all prosomal appendages, and ecologically mostly by the elevation in which it occurs, where *P. urku* occurs at an elevation almost twice that of *P. intag* sp. nov.

## Supplementary Material

XML Treatment for Paratropis

XML Treatment for Paratropis barragani

XML Treatment for Paratropis intag

## Additional information

### Use of AI

No use of AI was reported.

### Author ORCIDs

Joaquim Rodrigo Moraes de Castro de Castro https://orcid.org/0009-0006-3163-901X

### Data availability

All of the data that support the findings of this study are available in the main text or Supplementary Information.
